# Red cell distribution width improves the simplified acute physiology score for risk prediction in unselected critically ill patients

**DOI:** 10.1186/cc11351

**Published:** 2012-05-18

**Authors:** Sabina Hunziker, Leo A Celi, Joon Lee, Michael D Howell

**Affiliations:** 1 Department of Medicine, Division of Pulmonary, Critical Care, and Sleep Medicine, Beth Israel Deaconess Medical Center, 330 Brookline Avenue, Boston, MA 02115, USA; 2 Silverman Institute for Healthcare Quality and Safety, Beth Israel Deaconess Medical Center, 330 Brookline Avenue, Boston MA 02115, USA; 3 Laboratory for Computational Physiology, Harvard-MIT Division of Health Sciences and Technology, 200 Technology Square, Cambridge, MA 01239, USA; 4 Harvard Medical School, 260 Longwood Avenue, Boston, MA 02115, USA

## Abstract

**Introduction:**

Recently, red cell distribution width (RDW), a measure of erythrocyte size variability, has been shown to be a prognostic marker in critical illness. The aim of this study was to investigate whether adding RDW has the potential to improve the prognostic performance of the simplified acute physiology score (SAPS) to predict short- and long-term mortality in an independent, large, and unselected population of intensive care unit (ICU) patients.

**Methods:**

This observational cohort study includes 17,922 ICU patients with available RDW measurements from different types of ICUs. We modeled the association between RDW and mortality by using multivariable logistic regression, adjusting for demographic factors, comorbidities, hematocrit, and severity of illness by using the SAPS.

**Results:**

ICU-, in-hospital-, and 1-year mortality rates in the 17,922 included patients were 7.6% (95% CI, 7.2 to 8.0), 11.2% (95% CI, 10.8 to 11.7), and 25.4% (95% CI, 24.8 to 26.1). RDW was significantly associated with in-hospital mortality (OR per 1% increase in RDW (95%CI)) (1.14 (1.08 to 1.19), *P *< 0.0001), ICU mortality (1.10 (1.06 to 1.15), *P *< 0.0001), and 1-year mortality (1.20 (95% CI, 1.14 to 1.26); *P *< 0.001). Adding RDW to SAPS significantly improved the AUC from 0.746 to 0.774 (*P *< 0.001) for in-hospital mortality and 0.793 to 0.805 (*P *< 0.001) for ICU mortality. Significant improvements in classification of SAPS were confirmed in reclassification analyses. Subgroups demonstrated robust results for gender, age categories, SAPS categories, anemia, hematocrit categories, and renal failure.

**Conclusions:**

RDW is a promising independent short- and long-term prognostic marker in ICU patients and significantly improves risk stratification of SAPS. Further research is needed the better to understand the pathophysiology underlying these effects.

## Introduction

Red cell distribution width (RDW) is a measure of erythrocyte size variability and has been shown to be a prognostic marker for mortality, mainly in patients with cardiovascular disease and in community-dwelling patients, as well as in general in-hospital patients [1-16]. Although the mechanisms linking RDW to adverse patient outcomes remain incompletely understood, potential pathways include chronic inflammation [17,18], malnutrition [9-11], and anemia of different etiologies [19,20], among others. The prognostic potential of RDW is of particular interest because it is routinely included in the automated complete blood count (CBC) analyses in hospitalized patients and thus available at no additional cost for clinicians. Recent studies have found RDW to be a prognostic marker for short- and long-term mortality in critically ill patients [21-23]. The first study in critical illness was conducted in a cohort of 602 patients in China and found that RDW is associated with ICU mortality [22]. Recently, a large 10-year retrospective study from two US centers validated these findings and found RDW to be a robust predictor of the risk of all-cause patient mortality and bloodstream infection in the critically ill [21]. Finally, one report found RDW to be a strong outcome predictor in patients with pneumonia [23]. However, from these studies, it remains unclear whether RDW may improve state-of-the-art risk prediction in unselected critically ill patients.

We therefore aimed to investigate whether adding RDW has the potential to improve the prognostic performance of the Simplified Acute Physiology Score (SAPS) to predict short- and long-term mortality in an independent, large, and unselected population of ICU patients.

## Materials and methods

### Data source

This observational study used the prospectively collected Multiparameter Intelligent Monitoring in Intensive Care (MIMIC II) database, a publicly available clinical database developed by the Massachusetts Institute of Technology, Phillips Healthcare, and Beth Israel Deaconess Medical Center (BIDMC) since 2001 [24]. This database is a repository of de-identified physiologic, laboratory, and survival outcome data from more than 30,000 critically ill patients treated in ICUs at BIDMC. These data include clinical variables such as demographics (patient age, gender), highly granular physiologic data captured by the bedside monitors, medications administered and procedures performed, chronic disease diagnoses as represented by International Classification of Diseases (ICD)-9 codes, as well as laboratory results, such as complete blood count, serum chemistries, and microbiologic data. It further includes severity of illness, as assessed with SAPS I, and survival data within both the ICU and the hospital. The SAPS-I score was chosen in MIMIC II for its simplicity, requiring only available clinical laboratory measurements, fluid balance, and vital signs [25]. Finally, survival outcome data after hospital discharge is provided from the Social Security database. Patients included in this analysis were hospitalized between January 2001 and December 2008.

MIMIC II is a public de-identified ICU database that was developed with funding from the National Institutes of Health and the National Institute of Bio-imaging and Bioengineering. The project had approval from by Institutional Review Boards of both Beth Israel Deaconess Medical Center (Boston, MA, USA) and the Massachusetts Institute of Technology (Cambridge, MA, USA). The requirement for individual patient consent was waived because the study did not affect clinical care, and all protected health information had been deleted.

### Patients, outcomes, and covariates

We included all adult patients admitted to ICU floors who had an RDW measured on admission. RDW is routinely measured in admitted patients reported on every CBC and required no separate physician order. All CBCs were conducted on Advia Equipment (Siemens, models 2120 and 120). The normal reference range for RDW at our center is 10.5% to 15.5%. The primary outcome of interest was in-hospital mortality, defined as death before hospital discharge for any reason. The secondary outcomes of interest were ICU mortality, defined as death before ICU discharge and 1-year long-term mortality. The primary predictor of interest was RDW measured on ICU admission. We classified patients' comorbidity status based on the method described by Elixhauser *et al*. [26], as implemented by the Agency for Healthcare Research and Quality Comorbidity Software [27].

### Statistical analysis

First, we used descriptive statistics including mean with standard deviation and frequencies to describe the population, as appropriate. Next, we investigated the association between RDW used as a continuous variable and mortality by using logistic regression analysis and reported odds ratios (ORs) and 95% confidence intervals (CIs). We conducted univariable analysis and a multivariable analysis adjusted for SAPS, age, gender, hematocrit, and different comorbidities (anemia, renal failure, CHF, cardiac arrhythmias, valvular disease, chronic pulmonary disease, lung embolism, pneumonia, peripheral vascular disease, hypertension, paralysis, other neurologic diseases, diabetes, hypothyroidism, liver disease, peptic ulcer, HIV, lymphoma, cancer, rheumatoid arthritis, coagulopathy, obesity, weight loss, electrolyte disturbance, alcohol abuse, drug abuse, psychosis, and depression). To account for potential clustering within ICUs, we performed hierarchic modeling with a generalized estimating equation (GEE) by using an exchangeable variance-covariance structure with compound symmetry. Model discrimination was assessed by calculation of the receiver-operating characteristic (ROC) curves and reporting of the area under the curve (AUC). To assess the fit of the GEE model, we used the Quasi-likelihood under the Independence model Criterion (QIC) statistic, as previously suggested [28]. We also tested for effect modification by using interaction terms, to determine whether the effect of RDW on mortality varied by subgroups.

In addition to ROC analysis, we assessed the effect of adding RDW to risk-adjusted models by using reclassification-analysis methods, as suggested by Pencina [29]. Reclassification analysis is a more modern method that is widely recommended for assessing the incremental contribution of biomarkers to risk prediction. It is also more intuitive than areas under the ROC curve: for example, a net reclassification improvement (NRI) for mortality of 10% means that 10% of patients are more accurately classified by the new algorithm when compared with the old. The analyses used continuous variable information with evaluation of the effects on risk-category reclassification for survivors and nonsurvivors. This approach separately analyzed the reclassification of persons who died and those who did not die during the ICU and hospital course. Reclassification to a higher-risk group was considered upward movement in classification for nonsurvivors. Conversely, reclassification downward was considered a failure for nonsurvivors and vice versa for survivors. Improvement in reclassification was estimated by taking the sum of differences in proportions of individuals reclassified upward minus the proportion reclassified downward for nonsurvivors and the proportion of individuals moving downward minus the proportion moving upward for survivors. For estimating meaningful *a priori *mortality risk categories, we defined probabilities for mortality as < 2%, 2% to 5%, > 5% to 10%, > 10% to 20%, > 20% to 50%, and > 50%. We calculated the net reclassification improvement (NRI), which assesses improvement in reclassification over risk categories; we also assessed integrated discrimination improvement (IDI), which can be viewed as a continuous version of the NRI without the recourse to *a priori *defined risk categories.

All reported CIs are two-sided 95% intervals, and tests were performed at the two-sided 5% significance level. All analyses were performed with SAS 9.2.

## Results

### Population

We included a total of 17,922 patients admitted to one of five ICUs into this analysis. The mean age of patients was 63.2 years, and 57% were males. In total, 36% were medical ICU patients, 26% were surgical ICU patients, 16% were in the cardiac care unit, and 21% were from the cardiothoracic surgery unit. Baseline characteristics of the study population are presented in Table 1. Mean (standard deviation) RDW values in the overall cohort were 14.6% (± 2.0%).

**Table 1:** Characteristics of ICU cohort (*n *= 17,922)

Demographics	
Age (year), mean, median (SD, IQR)	63.2, 65.2 (± 17.6, 51.3 to 77.6)
Female gender, *n *(%)	7,717 (43.10%)
Type of ICU, *n *(%)	
Cardiac Care Unit	2,918 (16.28%)
Cardiothoracic Surgery Unit	3,824 (21.34%)
Medical Intensive Care Unit	6,536 (36.46%)
Surgical Intensive Care Unit	4,644 (25.91%)
Lab values on admission	
Hematocrit (%), mean, median (SD, IQR)	32.3, 32.0 (± 5.6, 28.4 to 36.0)
SAPS on admission, mean, median (SD, IQR)	13.6, 13.0 (± 5.5, 10.0 to 17.0)
Comorbidities, *n *(%)	
Anemia	4,225 (23.57%)
Chronic renal failure	3,641 (20.32%)
Congestive heart failure	3,301 (18.42%)
Peripheral vascular disease	1,347 (7.52%)
COPD	2,820 (15.73%)
Any cancer	2,887 (16.10%)
Diabetes	4,071 (22.71%)

COPD, chronic obstructive pulmonary disease; IQR, interquartile range; SAPS, Simplified Acute Physiology Score; SD, standard deviation.

### RDW and in-hospital and ICU mortality

**Figure 1:**
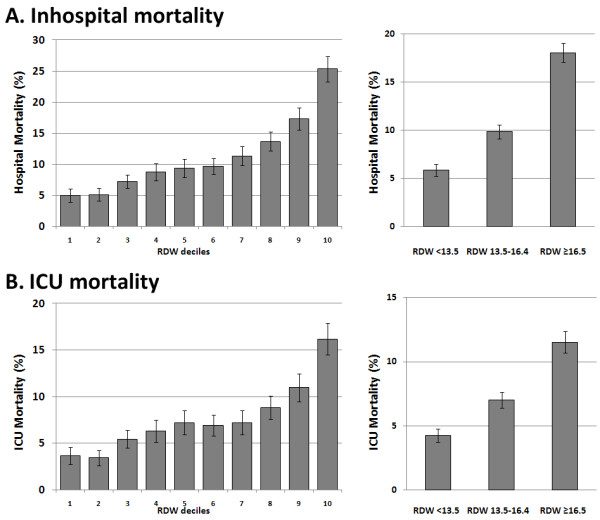
**Mortality**. Hospital mortality **(A) **and ICU mortality **(B) **within RDW deciles (left panel) and RDW categories (right panel).

**Figure 2:**
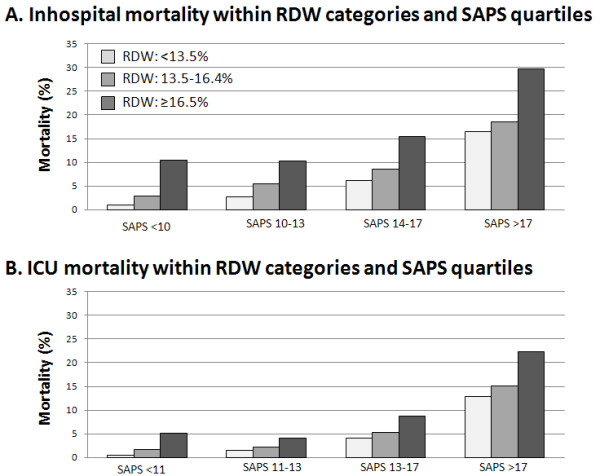
**RDW categories (< 13.5%, 13.5% to 16.4%, ≥16.5%) and mortality within SAPS subgroups**.

The in-hospital mortality rate was 11.2% (95% CI, 10.8 to 11.7), and ICU mortality was 7.6% (95% CI, 7.2 to 8.0). For graphic display, we divided RDW into deciles and plotted mortality within RDW deciles. Both in-hospital and ICU mortality increased with increasing RDW deciles, as displayed in Figure 1A and 1B (left panel). In-hospital mortality was around 5% in the lowest three deciles, increased to around 10% in deciles 4 to 7, and was around 20% in the three highest deciles. Based on this analysis, we defined three RDW categories: < 13.5%, 13.5% to 16.4%, and ≥16.5%, which approximately correspond to tertiles and in which mortality was low, intermediate, and high (Figure 1, right panel). Analyses stratified by severity of illness showed that these categories also predicted mortality rates within SAPS quartiles (Figure 2).

### Regression models for short-term mortality

**Figure 3:**
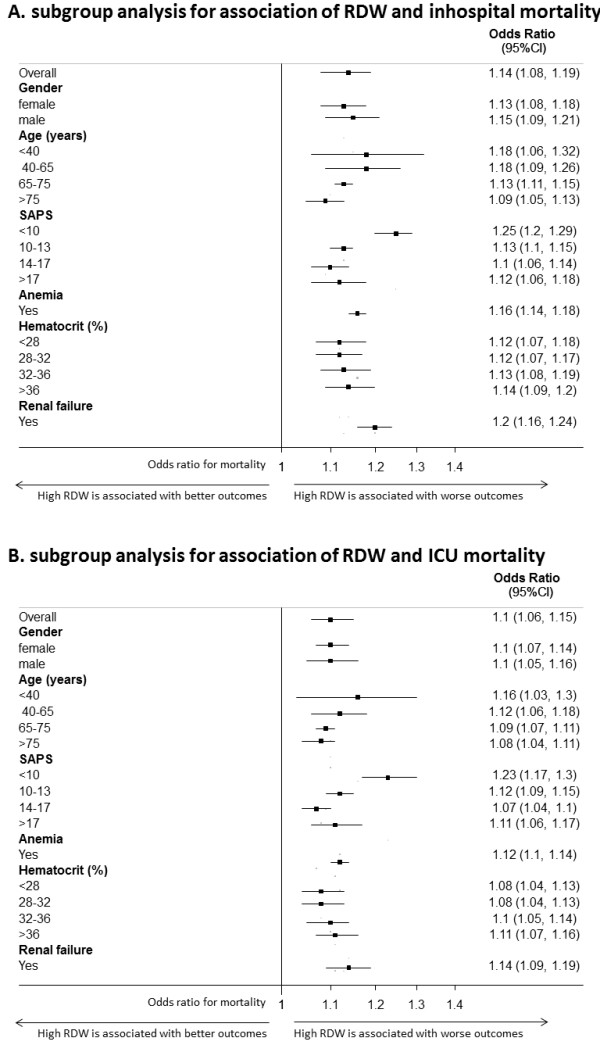
**(A) Subgroup analysis for association of RDW and in-hospital mortality**. **(B) **Subgroup analysis for association of RDW and ICU mortality.

With hierarchic modeling, we found that RDW used as a continuous variable was significantly associated with both in-hospital and ICU mortality. The association remained significant after adjusting for SAPS, age, gender, hematocrit, and different comorbidities, including anemia, in a multivariable model (Table 2). Of note, the odds ratio corresponds to a 1-unit (1%) increase in RDW. In both models, RDW was a significant outcome predictor independent of SAPS. We further analyzed the association between RDW and mortality in different predefined subgroups: gender, different age categories, SAPS quartiles, anemia, hematocrit quartiles, and renal failure. Figure 3 shows estimates for the different subgroups. Of note, the results proved to be robust across the different subgroups, particularly also in anemia patients and patients with different hematocrit values.

**Table 2:** Results from regression analysis

	In-hospital mortality		ICU mortality	
	**OR (95% CI)**	***P***	**OR (95% CI)**	***P***

Univariable				
RDW per 1% increase	1.20 (1.16, 1.24)	< 0.0001	1.18 (1.14, 1.22)	< 0.0001
SAPS per 1-point increase	1.19 (1.15, 1.23)	< 0.0001	1.21 (1.18, 1.25)	< 0.0001

Multivariable				
RDW per 1% increase	1.14 (1.08, 1.19)	< 0.0001	1.10 (1.06, 1.15)	< 0.0001
SAPS per 1-point increase	1.16 (1.13, 1.18)	< 0.0001	1.20 (1.18, 1.22)	< 0.0001

The multivariable hierarchic model is adjusted for age, gender, hematocrit, and different comorbidities (anemia, renal failure, chronic heart failure, cardiac arrhythmias, valvular disease, chronic pulmonary disease, lung embolism, pneumonia, peripheral vascular disease, hypertension, paralysis, other neurologic diseases, diabetes, hypothyroidism, liver disease, peptic ulcer, HIV, lymphoma, cancer, rheumatoid arthritis, coagulopathy, obesity, weight loss, electrolyte disturbance, alcohol abuse, drug abuse, psychosis, depression).

### Effect on discrimination and reclassification

We also evaluated the incremental benefit of adding RDW to SAPS by calculating AUCs and reclassification statistics (Table 3). For in-hospital mortality, adding RDW to SAPS improved the AUC from 0.746 to 0.774 (*P *< 0.001). Reclassification statistics showed a significant improvement in NRI of 0.175 (*P *< 0.0001), corresponding to a 17.5% improvement in classification of patients. This was mainly due to better classification of nonsurvivors into lower-risk classes. Detailed results of reclassification tables are presented in Additional file 1 (hospital mortality) and Additional file 2 (ICU mortality). The results for ICU mortality were similar, and adding RDW to SAPS improved the AUC from 0.793 to 0.805 (*P *< 0.001) (Table 3, right side). Reclassification statistics showed an NRI 0.090 (*P *< 0.0001), corresponding to a 9% improvement in classification. Adding RDW improved both the classification of nonsurvivors into higher-risk classes and the classification of survivors into lower-risk classes.

**Table 3:** Reclassification results for in-hospital and ICU mortality

	Hospital mortality				ICU mortality			
	**SAPS**	**SAPS and RDW**	**SAPS versus SAPS/RDW**	***P***	**SAPS**	**SAPS and RDW**	**SAPS versus SAPS/RDW**	***P***

Model χ^2 ^(log likelihoods)	1,505	1,831	326	< 0.0001	1,539	1,664	124	< 0.0001
Area under the ROC curve	0.746	0.774	0.028	< 0.001	0.793	0.805	0.012	< 0.001

Events								
Upward reclassification			362				197	
No reclassification			1,214				947	
Downward reclassification			309				153	

No events								
Upward reclassification			1,532				1,376	
No reclassification			9,849				12,412	
Downward reclassification			4,046				2257	

Net reclassification improvement			0.175 (SE 0.015)	< 0.0001			0.090 (SE 0.049)	< 0.0001
			
Integrated sensitivity	0.20	0.23	0.021	< 0.0001	0.19	0.20	0.01	< 0.0001
Integrated specificity	0.099	0.096	-0.0026	< 0.0001	0.07	0.06	-0.0008	< 0.0001
Integrated discrimination improvement			0.023 (SE 0.001)	< 0.0001			0.011 (SE 0.001)	< 0.0001

### RDW and 1-year survival

**Figure 4:**
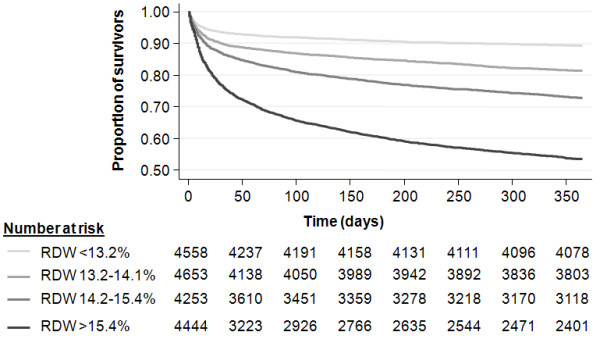
**Kaplan-Meier plot for 1-year survival within RDW quartiles (< 13.2%, 13.2% to 14.1%, 14.1% to 15.4%, and > 15.4%)**.

Finally, we evaluated the prognostic value of RDW to predict 1-year mortality within this ICU cohort. RDW was significantly associated with 1-year mortality in our multivariable adjusted model (OR, 1.20 (95% CI, 1.14 to 1.26); *P *< 0.001) and showed high discrimination of 1-year survivors and nonsurvivors (AUC, 0.73; 95% CI, 0.72 to 0.74). Figure 4 shows a Kaplan-Meier plot stratified by RDW quartiles to illustrate long-term survival rates.

## Discussion

In this large cohort of ICU patients, RDW strongly and independently predicted hospital mortality, ICU mortality, and 1-year mortality. As evidenced by improvement in both AUC and net reclassification index, RDW materially improved the prediction of mortality over and above traditional severity of illness, as measured with SAPS. This finding was robust across subgroups (for example, different age and hematocrit categories and comorbidities such as anemia or renal failure, factors that are known to be positively associated with RDW values [12,19,20]).

These results are in accordance with previous research showing an association of RDW and long-term mortality risks in different patient populations, such as patients with cardiovascular disease, chronic heart failure, and acute kidney injury treated with continuous renal-replacement therapy [2-16,30]. Cohort studies using nationally representative samples of the US population (Third National Health and Nutrition Survey (NHANES III)) also reported an association of RDW with long-term outcomes in community-dwelling patients [9-11]. In addition, recent studies have focused on critically ill patients and found RDW to be a prognostic in this setting as well [21-23]. Importantly, our results based on a broad US patient population admitted to different types of ICUs, including medical, cardiac, surgical, and cardiothoracic surgery confirm and expand these previous findings and demonstrate that RDW is a strong outcome predictor and thereby improves risk prediction based on the well-established SAPS score. We found the association of RDW and outcome to be robust when focusing on hospital and 1-year mortality overall and in different subgroups, such as patients with anemia.

Great interest is expressed in finding new prognostic markers in critically ill patients in the ICU setting. Early prognostication potentially helps to improve triage decisions in terms of early discharge of patients, and also with regard to therapeutic interventions. A prognostic marker should be evaluated based on the information it provides, beyond information already available based on clinical information, such as the information incorporated in SAPS. The SAPS system was developed to measure severity of illness in ICU patients, and subsequent research has validated its use to predict outcome in ICU patients [31]. Within our large sample, SAPS showed reasonable discrimination with AUCs between 0.75 and 0.80. Still, adding RDW to the SAPS further improved its discriminatory ability for both hospital mortality and ICU mortality. This was further confirmed when calculating reclassification statistics. For in-hospital mortality, this was mainly due to better classification of nonsurvivors into lower-risk classes, whereas for ICU mortality, RDW improved classification of survivors and nonsurvivors. Importantly, from a cost perspective, it should be noted that RDW is routinely measured in most patients as part of the admission CBC analysis and thus is free of additional costs. It will important for future studies to assess whether more-advanced severity of illness scores (that is, APACHE IV, SAPS III) can still be improved by the addition of RDW.

Our results provide an important target for future translational research. Why is RDW associated with increased mortality? The underlying mechanism is still unclear and represents an important avenue for future "bedside-to-bench" research. RDW can be increased in anemia or hemoglobinopathies, hemolysis, or after blood transfusions [19,20]. Yet, in our study and in the literature, the association of RDW and mortality was independent of anemia, and no evidence was found for effect modification by anemia and hematocrit levels [9-11,13]. Another possible pathophysiologic explanation is that RDW is a surrogate of inflammation, which is known to increase RDW. Several studies found RDW to be associated with blood markers of inflammation, such as interleukin-6, C-reactive protein (CRP) [11], as well as impaired iron mobilization [17]. Also, oxidative stress has been shown to increase anisocytosis by disrupting erythropoiesis, and to alter blood cell membrane deformability and red blood cell circulation half-life, ultimately leading to increased RDW [9,11,19]. Again, previous research found that adjusting for inflammation (for example, CRP) did not substantially reduce the prognostic value of RDW, demonstrating its effect beyond these factors [9-11,22]. In our cohort, we were not able to adjust the analysis for inflammatory markers, as these are not routinely measured on admission.

RDW may also reflect the patient's degree of physiological reserve, one of three main determinants of clinical outcome, as suggested by Bion [32]. The physiological reserve represents cellular response to acute stress and the resultant tissue hypoxia. Ischemia activates cellular systems that would reduce oxygen demand and physiologic processes that would improve tissue oxygen delivery, such as increased production and release of mature red cells into the peripheral bloodstream. How well this process of reactive erythropoiesis is carried out under oxidative stress may represent the patient's ability to handle acute physiological insult. Release of large immature red cells with poor oxygen-binding capacity, which results in an increased RDW, implies suboptimal response to oxidative stress. This may explain why the association between RDW and clinical outcome is independent of the severity of acute illness as well as the degree of inflammation. It may well represent the genetic factors that determine how well the body withstands physiological stress, whether from a severe infection, multitrauma, burns, or acute pancreatitis.

Strengths of this study are the large number of patients across different types of ICUs and the availability of a rich annotation of the physiological and clinical context, which allowed rigorous adjustment for severity of acute illness and subgroup analyses with high statistical power. We also included 1-year survival data on all the patients.

This study has limitations; as it was a retrospective, observational study, we were not able to assess causes for mortality and/or for elevated RDW. Also, our data provide no additional information about inflammation and oxidative stress. These pieces of information would be necessary to understand better the pathophysiology underlying the observed effects. Also, only admission RDW levels were considered, and it remains unclear whether changes over time of RDW may provide additional prognostic information. Importantly, we used SAPS, and more recent severity-of-illness scores (such as SAPS III or APACHE IV) have shown superior accuracy for outcome prediction; thus, future studies should address whether RDW has the potential also to improve these severity-of-illness scores. We calculated multivariable regression models, including important demographics and different comorbidities, to investigate the independent association of RDW and outcome; yet, co-linearity and missing clinical information on some important patient-history items (that is, transfusion) may limit the interpretation of the models.

## Conclusions

This analysis validated previous studies and found RDW to be a promising independent short- and long-term prognostic marker in ICU patients, which significantly improved the SAPS for risk stratification of patients. This was also true within several subgroups, including patients with anemia. If confirmed in future studies and with updated severity-of-illness scores (such as SAPS III or APACHE IV), RDW may help to improve current prediction scores at little or no additional costs for improved decision-making in ICU patients. Further studies should be conducted to understand better the mechanism linking RDW to these adverse outcomes.

## Key messages

• Red cell distribution width (RDW), a measure of erythrocyte size variability, is a promising independent short- and long-term prognostic marker in ICU patients

• In this large U.S. cohort study including 17,922 patients from different types of ICUs, RDW was a strong and independent outcome predictor for ICU-, in-hospital, and 1-year mortality

• RDW further improved the prognostic accuracy of the SAPS score

## Abbreviations

AUC: Area under the curve; BIDMC: Beth Israel Deaconess Medical Center; COPD: chronic obstructive pulmonary disease; CBC: complete blood count; CI: confidence interval; GEE: generalized estimating equation; IDI: integrated discrimination improvement; ICD: International Classification of Diseases; IQR: interquartile range; MIMIC II: Multiparameter Intelligent Monitoring in Intensive Care; NRI: net reclassification improvement; OR: odds ratio; QIC: Quasi-likelihood under the Independence model Criterion; ROC: receiver-operating characteristic curves; RDW: red cell distribution width; SAPS: Simplified Acute Physiology Score; SD: standard deviation.

## Competing interests

The authors declare that they have no competing interests.

## Authors' contributions

SH, LAC, JL, and MH conceived and designed the study and wrote the study protocol. JL and LAC extracted data. The statistical analyses and the first draft of manuscript were performed by SH. All authors amended and commented on the manuscript and approved the final version.

## Supplementary Material

Additional file 1 **Reclassification for in-hospital mortality**. Reclassification table for hospital mortality prediction in different *a priori *risk strata; upper table is for hospital survivors; lower table is for hospital nonsurvivors. Click here for file

Additional file 2 **Reclassification for ICU mortality**. Reclassification table for ICU mortality prediction in different *a priori *risk strata; upper table is for ICU survivors; lower table is for ICU nonsurvivors. Click here for file
