# Supplementary material for: Red cell distribution width improves the simplified acute physiology score for risk prediction in unselected critically ill patients
Source: Crit Care. 2012 May 18;16(3):R89. doi: 10.1186/cc11351 (PMC3580634; doi:10.1186/cc11351)
Supplement: Additional file 1 — Reclassification for in-hospital mortality. Reclassification table for hospital mortality prediction in different a priori risk strata; upper table is for hospital survivors; lower table is for hospital nonsurvivors. [file cc11351-S1.DOC]

**Additional file 1: Reclassification for inhospital mortality**

Reclassification table for hospital mortality prediction in different a priori risk strata; upper table is for hospital survivors, lower table is for hospital non-survivors

| **Model with SAPS only** | **Combined model with SAPS and RDW** | | | | | | |
| --- | --- | --- | --- | --- | --- | --- | --- |
| **Hospital survivors** | | | | | | | |
| ***risk*** | *<2%* | *2-5%* | *5-10%* | *>10-20%* | *20-50%* | *>50%* | **Total** |
| <2% | 973 | 84 | 9 | 0 | 1 | 0 | 1067 |
|  | 6.3 | 0.5 | 0.1 | 0.0 | 0.0 | 0.0 | 6.9 |
| 2-5% | 788 | 2884 | 417 | 59 | 5 | 1 | 4154 |
|  | 5.1 | 18.7 | 2.7 | 0.4 | 0.0 | 0.0 | 26.9 |
| 5-10% | 0 | 1315 | 2373 | 437 | 49 | 2 | 4176 |
|  | 0.0 | 8.5 | 15.4 | 2.8 | 0.3 | 0.0 | 27.1 |
| 5-10% | 0 | 0 | 1408 | 2439 | 392 | 9 | 4248 |
|  | 0.0 | 0.0 | 9.1 | 15.8 | 2.5 | 0.1 | 27.5 |
| 20-50% | 0 | 0 | 0 | 520 | 1146 | 67 | 1733 |
|  | 0.0 | 0.0 | 0.0 | 3.4 | 7.4 | 0.4 | 11.2 |
| >50% | 0 | 0 | 0 | 0 | 15 | 34 | 49 |
|  | 0.0 | 0.0 | 0.0 | 0.0 | 0.1 | 0.2 | 0.3 |
| **Total** | 1761 | 4283 | 4207 | 3455 | 1608 | 113 | 15427 |
|  | 11.4 | 27.8 | 27.3 | 22.4 | 10.4 | 0.7 | 100.0 |
| **Hospital nonsurvivors** | | | | | | | |
| ***risk*** | *<2%* | *2-5%* | *5-10%* | *>10-20%* | *20-50%* | *>50%* | **Total** |
| <2% | 6 | 2 | 1 | 0 | 0 | 0 | 9 |
|  | 0.3 | 0.1 | 0.1 | 0.0 | 0.0 | 0.0 | 0.5 |
| 2-5% | 4 | 75 | 37 | 18 | 1 | 0 | 135 |
|  | 0.2 | 3.9 | 1.9 | 0.9 | 0.1 | 0.0 | 7.1 |
| 5-10% | 0 | 62 | 217 | 82 | 12 | 0 | 373 |
|  | 0.0 | 3.2 | 11.3 | 4.3 | 0.6 | 0.0 | 19.5 |
| 5-10% | 0 | 0 | 125 | 377 | 126 | 11 | 639 |
|  | 0.0 | 0.0 | 6.5 | 19.7 | 6.6 | 0.6 | 33.4 |
| 20-50% | 0 | 0 | 0 | 117 | 452 | 72 | 641 |
|  | 0.0 | 0.0 | 0.0 | 6.1 | 23.6 | 3.8 | 33.5 |
| >50% | 0 | 0 | 0 | 0 | 31 | 87 | 118 |
|  | 0.0 | 0.0 | 0.0 | 0.0 | 1.6 | 4.5 | 6.2 |
| **Total** | 10 | 139 | 380 | 594 | 622 | 170 | 1915 |
|  | 0.5 | 7.3 | 19.8 | 31.0 | 32.5 | 8.9 | 100.0 |
