# Supplementary material for: Red cell distribution width improves the simplified acute physiology score for risk prediction in unselected critically ill patients
Source: Crit Care. 2012 May 18;16(3):R89. doi: 10.1186/cc11351 (PMC3580634; doi:10.1186/cc11351)
Supplement: Additional file 2 — Reclassification for ICU mortality. Reclassification table for ICU mortality prediction in different a priori risk strata; upper table is for ICU survivors; lower table is for ICU nonsurvivors. [file cc11351-S2.DOC]

**Additional file 2: Reclassification for ICU mortality**

Reclassification table for ICU mortality prediction in different a priori risk strata; upper table is for ICU survivors, lower table is for ICU non-survivors

| **Model with SAPS only** | **New model with SAPS and RDW** | | | | | | |
| --- | --- | --- | --- | --- | --- | --- | --- |
| **ICU survivors** | | | | | | | |
| ***risk*** | *<2%* | *2-5%* | *5-10%* | *>10-20%* | *20-50%* | *>50%* | **Total** |
| <2% | 3915 | 296 | 10 | 0 | 0 | 0 | 4221 |
|  | 24.4 | 1.8 | 0.1 | 0.0 | 0.0 | 0.0 | 26.3 |
| 2-5% | 938 | 3927 | 543 | 31 | 3 | 0 | 5442 |
|  | 5.9 | 24.5 | 3.4 | 0.2 | 0.0 | 0.0 | 33.9 |
| 5-10% | 0 | 447 | 2193 | 257 | 14 | 0 | 2911 |
|  | 0.0 | 2.8 | 13.7 | 1.6 | 0.1 | 0.0 | 18.1 |
| 5-10% | 0 | 0 | 664 | 1688 | 194 | 1 | 2547 |
|  | 0.0 | 0.0 | 4.1 | 10.5 | 1.2 | 0.0 | 15.9 |
| 20-50% | 0 | 0 | 0 | 188 | 650 | 27 | 865 |
|  | 0.0 | 0.0 | 0.0 | 1.2 | 4.1 | 0.2 | 5.4 |
| >50% | 0 | 0 | 0 | 0 | 20 | 39 | 59 |
|  | 0.0 | 0.0 | 0.0 | 0.0 | 0.1 | 0.2 | 0.4 |
| **Total** | 4853 | 4670 | 3410 | 2164 | 881 | 67 | 16045 |
|  | 30.3 | 29.1 | 21.3 | 13.5 | 5.5 | 0.4 | 100.0 |
| **ICU nonsurvivors** | | | | | | | |
| ***risk*** | *<2%* | *2-5%* | *5-10%* | *>10-20%* | *20-50%* | *>50%* | **Total** |
| <2% | 32 | 4 | 0 | 0 | 0 | 0 | 36 |
|  | 2.5 | 0.3 | 0.0 | 0.0 | 0.0 | 0.0 | 2.8 |
| 2-5% | 13 | 146 | 54 | 2 | 0 | 0 | 215 |
|  | 1.0 | 11.3 | 4.2 | 0.2 | 0.0 | 0.0 | 16.6 |
| 5-10% | 0 | 16 | 166 | 48 | 7 | 0 | 237 |
|  | 0.0 | 1.2 | 12.8 | 3.7 | 0.5 | 0.0 | 18.3 |
| 5-10% | 0 | 0 | 54 | 255 | 62 | 0 | 371 |
|  | 0.0 | 0.0 | 4.2 | 19.7 | 4.8 | 0.0 | 28.6 |
| 20-50% | 0 | 0 | 0 | 41 | 269 | 20 | 330 |
|  | 0.0 | 0.0 | 0.0 | 3.2 | 20.7 | 1.5 | 25.4 |
| >50% | 0 | 0 | 0 | 0 | 29 | 79 | 108 |
|  | 0.0 | 0.0 | 0.0 | 0.0 | 2.2 | 6.1 | 8.3 |
| **Total** | 45 | 166 | 274 | 346 | 367 | 99 | 1297 |
|  | 3.5 | 12.8 | 21.1 | 26.7 | 28.3 | 7.6 | 100.0 |
